# Leukemia inhibitory factor via the Toll-like receptor 5 signaling pathway involves aggravation of cachexia induced by human gastric cancer-derived 85As2 cells in rats

**DOI:** 10.18632/oncotarget.26190

**Published:** 2018-10-05

**Authors:** Kiyoshi Terawaki, Yohei Kashiwase, Miaki Uzu, Miki Nonaka, Yumi Sawada, Kanako Miyano, Yoshikazu Higami, Kazuyoshi Yanagihara, Masahiro Yamamoto, Yasuhito Uezono

**Affiliations:** ^1^ Division of Cancer Pathophysiology, National Cancer Center Research Institute, Chuo-Ku, Tokyo 104-0045, Japan; ^2^ Tsumura Kampo Research Laboratories, Kampo Research & Development Division, Tsumura & Co., Inashiki-Gun, Ibaraki 300-1192, Japan; ^3^ Laboratory of Molecular Pathology and Metabolic Disease, Faculty of Pharmaceutical Sciences, Tokyo University of Science, Noda, Chiba 278-8510, Japan; ^4^ Division of Biomarker Discovery, Exploratory Oncology Research & Clinical Trial Center, National Cancer Center, Chiba 277-8577, Japan; ^5^ Division of Supportive Care Research, Exploratory Oncology Research & Clinica l Trial Center, National Cancer Center, Chuo-Ku, Tokyo 104-0045, Japan; ^6^ Innovation Center for Supportive, Palliative and Phychosocial Care, National Cancer Center Hospital, Chuo-Ku, Tokyo 104-0045, Japan

**Keywords:** cancer cachexia, anorexia, gastric cancer, leukemia inhibitory factor, toll-like receptor

## Abstract

Cancer cachexia is highly prevalent in gastric cancer patients and characterized by decreased food consumption and body weight. We previously created a rat model of cancer cachexia using MKN45cl85 and 85As2 cells derived from human gastric cancer. The 85As2 cells induced cachexia more potently compared to MKN45cl85 cells. To clarify the mechanism underlying the difference in the cachexia-inducing ability of these cells, we conducted DNA microarray analysis, focusing on cell proliferation and the production of leukemia inhibitory factor (LIF), a cachexia-inducing factor. The plasma human LIF levels of 85As2-induced cachexic rats increased as symptoms worsened, whereas the plasma levels of MKNcl85 were low. 85As2 cells displayed more genetic changes compared to MKN45cl85 cells, which were related to Toll-like receptor (TLR) 4/5 signaling. Stimulation of both cells with TLR4 (lipopolysaccharide) or TLR5 (flagellin) agonists did not affect proliferation. However, in 82As2 cells, LIF production was significantly increased by stimulation with TLR5, which was suppressed by an inhibitor of interleukin-1 receptor-associated kinase-1/4, which are important factors in the TLR5 signaling pathway. The increase in LIF production resulting from activation of the TLR5 signaling pathway may contribute to the cachexia-inducing ability of 85As2 cells.

## INTRODUCTION

Cancer cachexia is characterized by a decrease in body weight and food consumption and occurs in 80% of patients with progressive cancer, causing at least 20% of cancer-related deaths [1–3]. The European Palliative Care Research Collaborative guidelines define cancer cachexia as “a multi-factorial syndrome defined by an ongoing loss of skeletal muscle mass (with or without loss of fat mass) that cannot be fully reversed by conventional nutritional support and leads to progressive functional impairment. The pathophysiology is characterized by a negative protein and energy balance driven by a variable combination of reduced food intake and abnormal metabolism.” [4]. A diagnosis of cancer cachexia is given if three of the following five criteria are met, in addition to a decrease in body weight: anorexia, muscle weakness, decreased lean mass, fatigue, and abnormal values from biochemical tests (anemia, increased inflammatory markers, low albumin levels) [1]. The incidence of cancer cachexia differs depending on the cancer type. Specifically, gastric cancer has a remarkably high incidence (83%) of body weight decrease, as does pancreatic cancer. The incidence is lower in breast cancer, acute nonlymphocytic leukemia, and sarcomas [3]. Many cancer cachexia models have been established, but a suitable animal model of cachexia using human gastric cancer cells is not available [5, 6].

To establish an animal model of cancer cachexia using human gastric cancer cells, we previously screened 15 human gastric cancer cell lines using subcutaneous xenografts in mice, using a reduction in body weight as an indicator of cachexia. MKN-45 was the only cell line to cause a reduction in body weight (40% incidence). Through repeated subcutaneous xenografting and tumor collection from MKN-45 cells, we obtained a novel cell line causing body weight reduction in 100% of mice. The cell line was designated as MKN45cl85 [7]. MKN45cl85 cells were orthotopically grafted into mice, and peritoneal cavity cells were collected from specimens exhibiting peritoneal metastasis. These cells were then repeatedly cultured and xenografted. This established another cell line, 85As2, with a 100% incidence of body weight decrease and peritoneal metastasis in mice [7]. Furthermore, through subcutaneous xenografts of 85As2 cells, we established an experimental rat model of cachexia that meets the clinical diagnostic criteria of cachexia. This model may be useful for determining the mechanism of cachexia progression and treatment methods [8]. Interestingly, 85As2 cell xenografts in cachexic rats caused earlier and more severe cachexia symptoms than xenografts of the parent MKN45cl85 cells. However, the detailed mechanism of these differences in the cachexia-inducing ability of the two cell lines is unclear.

Toll-like receptors (TLRs) are receptor proteins on the surface of animal cells that recognize pathogens such as portions of viruses and bacteria and activate innate immunity. TLRs are pattern recognition receptors, of which 10 types exist in humans [9–12]. The TLR family of proteins is split into two main groups based on their intracellular localization. TLR1, 2, 4, 5, 6, and 10 are expressed on the cell surface and recognize sugars, lipids, and proteins on bacteria and viruses. Specifically, they recognize the epithelial components of microorganisms and transmit a signal from the cell surface into the cell. In contrast, TLR3, 7, 8, and 9 are expressed in the endoplasmic reticulum and endosomes inside cells and respond to pathogen-derived nucleic acids. The ligands recognized by TLRs are generally determined by the TLR subtype. For example, TLR4 recognizes lipopolysaccharide (LPS), which is a structural component of the outer membrane of gram-negative bacteria, TLR2 recognizes lipoproteins on gram-positive bacteria, TLR3 recognizes virus double-stranded RNA, TLR5 recognizes flagellin in cell flagella and an unknown pathogen-associated molecular pattern, and TLR9 recognizes non-methylated CpG islands in viral DNA [13–15]. Therefore, TLRs are important in host immunity and contribute to inflammatory cytokine production similarly to interleukin (IL)-1 and 6 and tumor necrosis factor-alpha (TNF-α) [12]. Recently, tumor proliferation and formation were shown to be related to TLRs [16, 17].

Infection with *Helicobacter pylori* is a known factor affecting the onset of gastric cancer. It has been suggested that a response of LPS in *H. pylori* to TLR 2, 4, and 5 is involved in the mechanism of onset [18–22]. Inflammatory cytokines play an important role in promoting tumor formation by TLRs. The roles of inflammatory cytokines such as IL-1, 6, TNF-α, and leukemia inhibitory factor (LIF) in causing cancer cachexia are known [23–26]. However, the detailed relationship between cancer cachexia and TLRs is unclear.

In our previous study, we suggested that human LIF is a causative factor in the 85As2-induced cachexia model [8]. Clarifying the mechanism of the difference in the cachexia-inducing ability between the parent MKN45cl85 cell line and 85As2 cells, which show an enhanced cachexia-inducing ability, may improve the understanding the mechanism of the onset or aggravation of cancer cachexia. Therefore, in the present study, we conducted DNA microarray analysis of 85As2 and MKN45cl85 cells to assess the mechanism causing the differences in cachexia-inducing ability. The results suggest that gene function changes between the two cell lines affect cancer cell growth and proliferation as well as tumor morphology. Furthermore, the results suggest that the TLR4/5 signaling pathway is activated in 85As2 cells. Thus, we conducted a detailed analysis focusing on cellular proliferation and LIF production to investigate how changes in TLR4/5 signaling affect the early expression and severity of cachexia symptoms in rats with 85As2 cell xenografts.

## RESULTS

### 85As2 cells induce more severe cachexia than MKN45cl85 cells

**Figure 1:**
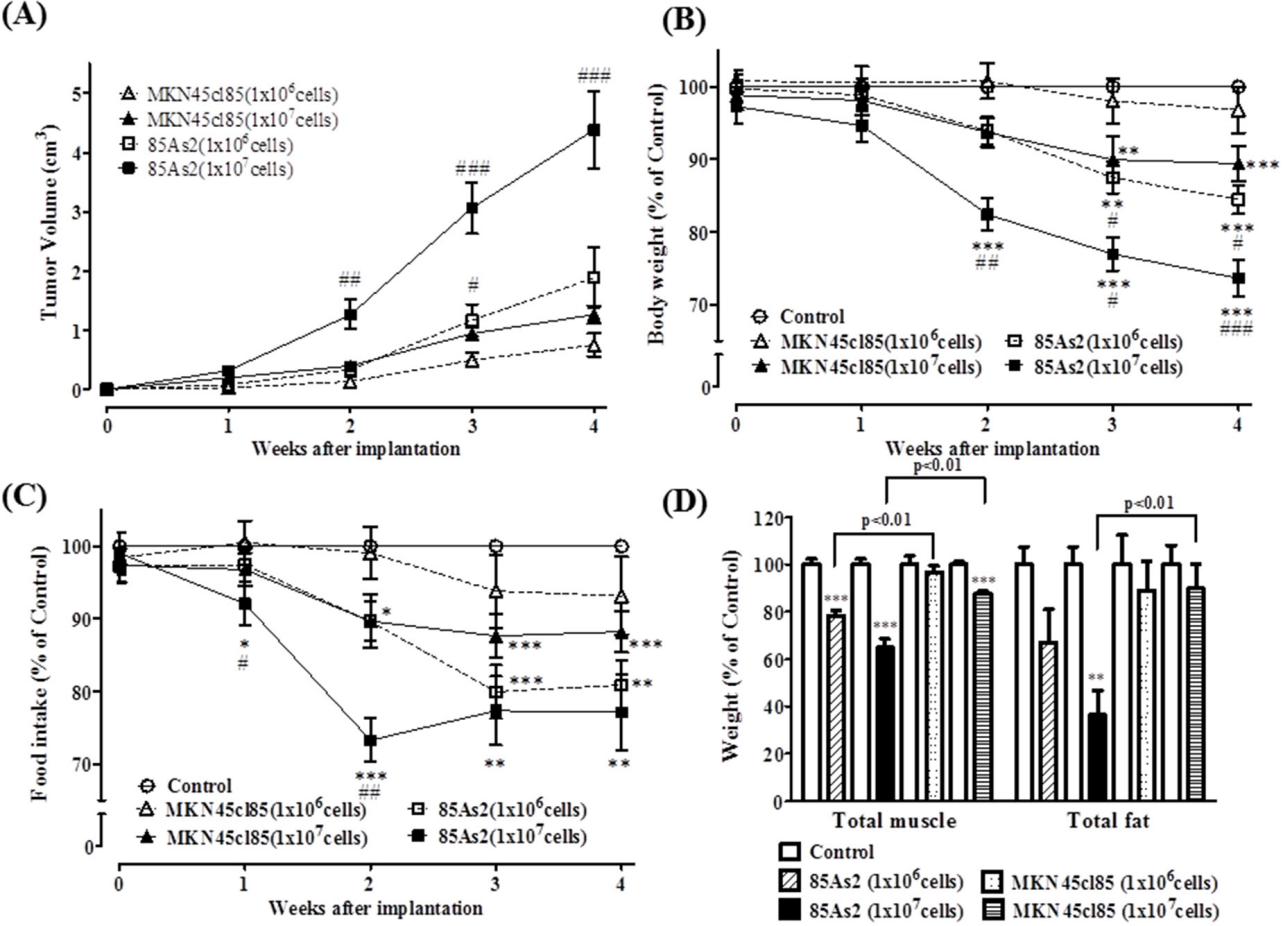
**(A)** Tumor volume, **(B)** body weight, **(C)** food intake, and **(D)** muscle and fat weight in the MKN45cl85- and 85As2-induced cancer cachexia groups 4 weeks after implantation of cells in nude rats. Rats were inoculated subcutaneously with MKN45cl85 or 85As2 cells in both flanks (1 × 10^6^ or 1 × 10^7^ cells per site) on week 0. Rats inoculated with saline served as the control group. Muscle tissues were expressed as the total weights of greater pectoral, gastrocnemius, tibialis, and soleus. Fat tissues were expressed as the total weights of epididymis, perirenal, and mesentery fat. The data for body weight, food intake, and muscle and fat weight were expressed as percentage (%) of control. Each data point represents the mean ± SEM of 9–10 rats. Each data point about MKN45cl85 (1 × 10^6^ cells) represents the mean ± SEM of five rats. Each column about muscle and fat weight represents the mean ± SEM of five rats. Differences between groups were evaluated using Aspin–Welch's *t*-test. ^*^p < 0.05, ^**^p < 0.01, ^***^p < 0.001 vs. the control group; ^#^p < 0.05, ^##^p < 0.01, ^###^p < 0.001 vs. the corresponding cell concentration MKN45cl85 group.

To compare the cachexia-inducing ability of MKN45cl85 and 85As2 cells, two cell concentrations (1 × 10^6^ or 1 × 10^7^ cells) were xenotransplanted subcutaneously on both sides of the abdomen in nude rats. Time-dependent and cell concentration-dependent tumor enlargement was observed in both cell xenograft groups (Figure 1A). The 85As2 cell xenograft group exhibited rapid tumor enlargement and markedly increased tumor volume. In contrast, the MKN45cl85 cell xenograft group exhibited moderate tumor enlargement. Over the same period, the rate of tumor was slower and tumor volume was smaller than in the 85As2 xenograft group. The 85As2 group showed a significantly larger tumor volume than the MKN45cl85 cell xenograft group in rats administered the same cell concentrations. Additionally, the use of luciferase-tagged MKN45cl85 and 85As2 cells indicated that cell proliferation in the tumor tissue was higher than that of MKN45cl85 cells (Supplementary Figure 1).

Body weight in the control group continually increased during the observation period (averages for all 25 control rats: 0 weeks, 192.7 ± 2.8 g; 4 weeks, 267.0 ± 3.9 g). Rats in the xenograft group inoculated with 1 × 10^6^ MKN45cl85 cells did not display a significant decrease in body weight compared to the control group (4 weeks: 1 × 10^6^ cells, 96.2 ± 2.5%, n = 5, vs. control, 267.1 ± 8.1 g; n = 5). The xenograft group inoculated with 1 × 10^7^ MKN45cl85 cells displayed a significant decrease in body weight after 3 weeks (88.0 ± 2.9%, n = 9 vs. control, 266.8 ± 6.1 g; n = 10) (Figure 1B). In contrast, the xenograft group inoculated with 1 × 10^6^ 85As2 cells showed decreased body weight compared to the control group, and the difference became significant after 3 weeks, with a value at 4 weeks of 84.4 ± 2.0% (n = 10) compared to the control group (267.1 ± 7.0 g; n = 10). Furthermore, the xenograft group inoculated with 85As2 1 × 10^7^ cells displayed a more prominent decrease in body weight. The decrease became significant at 2 weeks and continued to decrease until week 4 (73.7 ± 2.6%; n = 9 vs. control, 258.1 ± 4.9 g; n = 10). A significant difference was evident between the same concentrations of 85As2 and MKN45cl85 cells from 2 to 4 weeks (2 weeks: 1 × 10^7^ cells, p < 0.01; 3 weeks: 1 × 10^7^ cells, p < 0.05 and 1 × 10^6^ cells, p < 0.05; 4 weeks: 1 × 10^7^ cells, p < 0.001 and 1 × 10^6^ cells, p < 0.05).

Both the 85As2 1 × 10^6^ cell group (after 2 weeks) and 1 × 10^7^ cell group (after 1 week) displayed significantly lower food intake than the control group (4 weeks:1 × 10^7^ cells; 77.4 ± 5.2%; n = 9 vs. control, 20.1 ± 0.5 g; n = 10) (4 weeks:1 × 10^6^ cells; 80.7 ± 3.4%; n = 10 vs. control, 20.9 ± 0.5 g; n = 10) (Figure 1C). Similar to the results for body weight decrease, the xenograft group inoculated with 1 × 10^7^ MKN45cl85 cells displayed significantly lower food intake after 2 weeks (4 weeks: 87.9 ± 3.2%; n = 9 vs. control, 20.6 ± 0.5 g; n = 10). However, the xenograft group inoculated with 1 × 10^6^ MKN45cl85 cells did not display significantly lower food intake during the observation period (4 weeks: 93.0 ± 4.2%; n = 5, vs. control, 22.1 ± 0.7 g; n = 5). There was a significant difference between the xenograft groups inoculated with 1 × 10^7^ cells of 85As2 and MKN45cl85 after 1 week (p < 0.05) and 2 weeks (p < 0.01).

Additionally, after 4 weeks, we measured muscle and fat weight in rats with xenografts of both cell types. Similar to the results for body weight decrease, the xenograft groups inoculated with 1 × 10^6^ and 1 × 10^7^ 85As2 cells displayed significantly lower muscle weight than the control group (1 × 10^7^ cells: 64.8 ± 3.6%; n = 5 and 1 × 10^6^ cells: 78.1 ± 2.4%; n = 5 vs. control, 6.65 ± 0.16 g; n = 5) (Figure 1D). In contrast, the xenograft group inoculated with 1 × 10^7^ MKN45cl85 cells displayed significantly lower muscle weight (87.5 ± 1.1%; n = 5 vs. control, 6.98 ± 0.07 g; n = 5), while using 1 × 10^6^ cells in the xenografts did not significantly lower muscle weight (96.6 ± 2.7%; n = 5 vs. control, 6.65 ± 0.24 g; n = 5). Furthermore, there was a significant difference between the same concentrations of 85As2 and MKN45cl85 cells (p < 0.01 for both 1 × 10^6^ and 1 × 10^7^ cells). The 85As2 1 × 10^7^ cell group displayed significantly lower fat weight (36.3% ± 10.5%; n = 5) than the control group (7.77 ± 0.58 g; n = 5). There was also a significant difference between the same concentrations of 85As2 and MKN45cl85 cells (1 × 10^7^ cells: p < 0.01). The MKN45cl85 1 × 10^7^ cell xenograft group did not exhibit significantly lower fat weights.

### Plasma LIF levels in 85As2 cell-induced cachexia rat model are significantly higher than in MKN45cl85 model

**Figure 2:**
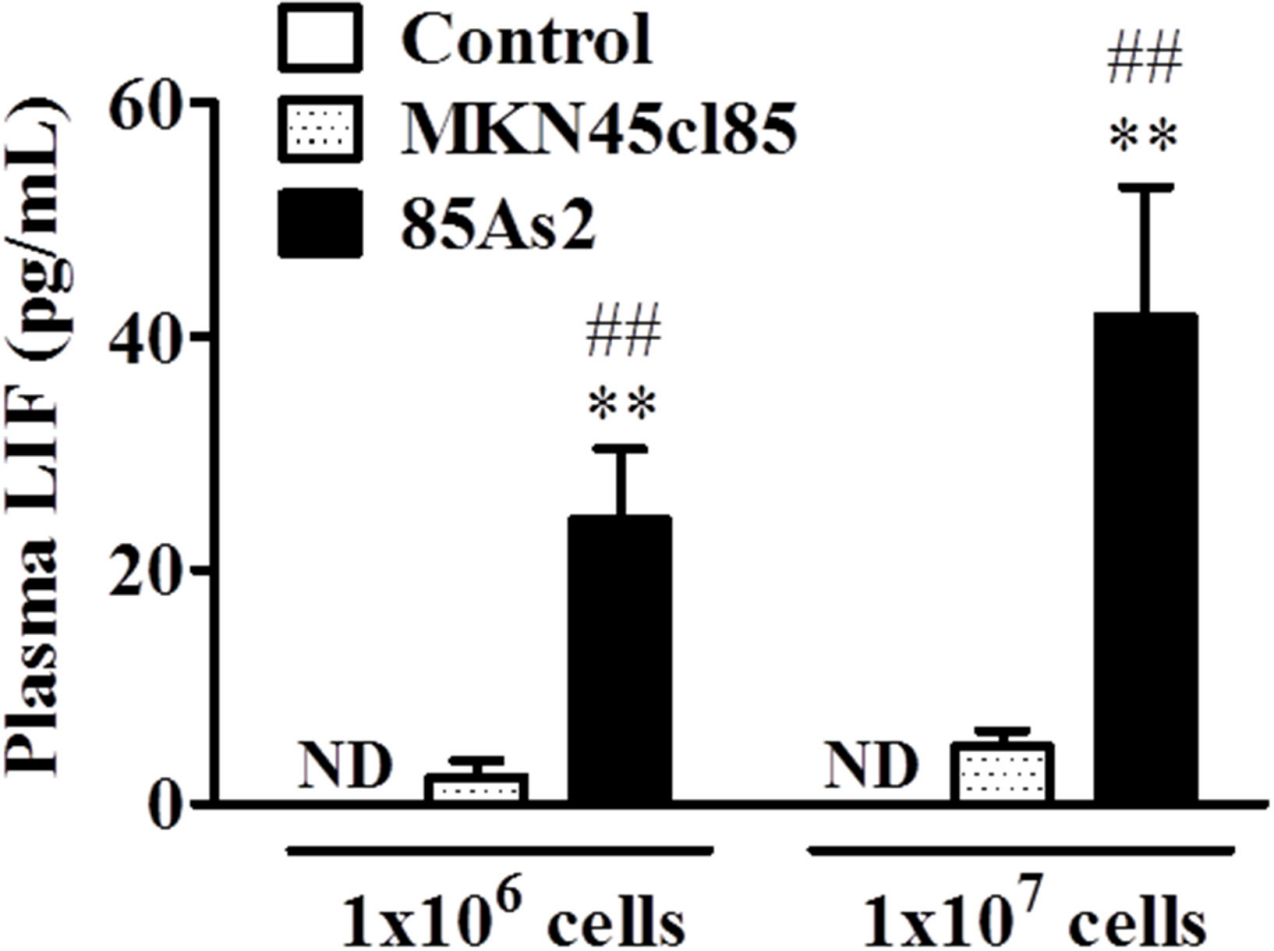
Plasma levels of human LIF in the MKN45cl85- or 85As2-implanted cancer cachexia rat model 4 weeks after implantation Rats were subcutaneously implanted with MKN45cl85 or 85As2 cells in both flanks (1 × 10^6^ or 1 × 10^7^ cells at each site) on week 0. Rats inoculated with saline served as the control group. Plasma LIF levels are expressed as the mean ± SEM (pg/mL) values for 4–5 rats. Plasma LIF levels in control rats were not detectable during the experiment. Differences in plasma LIF levels between groups were evaluated using the Kruskal-Wallis test followed by a post-hoc Dunn's multiple comparison test (^*^p < 0.05, ^**^p < 0.01 vs. the corresponding control group). Differences in LIF levels in cell implantation groups were evaluated using the Mann-Whitney U-test (^#^p < 0.05, ^##^p < 0.01 vs. the corresponding MKN45cl85 group). LIF: leukemia inhibitory factor; ND: not detectable (below the minimum detection limit of the assay).

After 4 weeks, we measured the plasma LIF concentration in rats with xenografts of both cell types and in the non-tumor bearing control group. The LIF concentration in the control group was below the detection limit. The plasma LIF concentration in the MKN45cl85 cell xenograft group was higher than that in the control group, but the increase was not significant at either cell concentration (Figure 2). The 85As2 cell xenograft group exhibited a significant and cell concentration-dependent increase in plasma LIF levels compared to the control group (p < 0.01 for both 1 × 10^6^ and 1 × 10^7^ cells). Furthermore, there was a significant difference between the same concentrations of 85As2 and MKN45cl85 cells (1 × 10^6^ cells: p < 0.01 vs. MKN45cl85; 1 × 10^7^ cells: p < 0.01 vs. MKN45cl85).

### IL-6 family inflammatory cytokine production in MKN45cl85 and 85As2 cells

**Figure 3:**
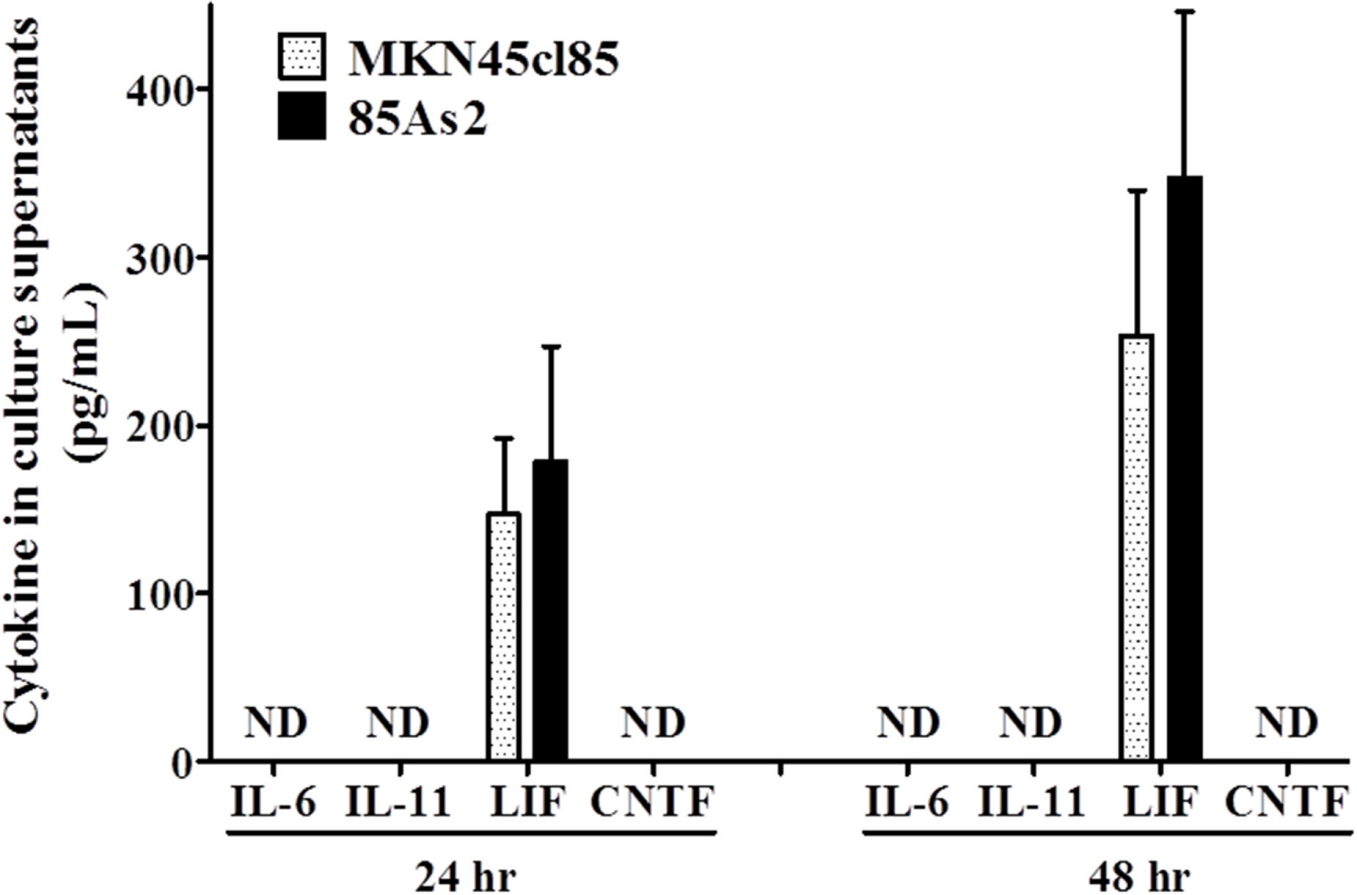
Levels of proinflammatory cytokines belonging to the IL-6 family including human LIF in cell culture supernatants from the MKN45cl85 or 85As2 cell line at 2. 5 × 10^5^ cells/well Cytokine levels are expressed as the mean ± SEM (pg/mL) values for the three experiments. Human IL-6, IL-11, and CNTF, but not LIF, were not detectable (below the minimum detection limit of the assay). LIF: leukemia inhibitory factor; IL-: interleukin-; CNTF: ciliary neurotrophic factor; ND: not detectable (below the minimum detection limit of the assay).

In addition to measuring LIF production in MKN45cl85 and 85As2 cells, we measured the culture supernatants of both cell groups to clarify the production of IL-6 family inflammatory of cytokines. Both cell groups were cultured for either 24 or 48 h. At each time point, both groups displayed prominent and similar levels of production of human LIF (Figure 3). In contrast, the IL-6 family of inflammatory cytokines (human IL-6, IL-11, ciliary neurotrophic factor [CNTF]) were below the detection limit in the culture supernatants obtained from both cell groups.

### Comparison of MKN45cl85 and 85As2 cells by DNA microarray analysis

DNA microarray analysis was performed using MKN45cl85 and 85As2 cells to clarify the underlying mechanism of the difference in their cachexia-inducing abilities. After normalization of the digitized array data, the numbers of valid probes for MKN45cl85 and 85As2 cells were 25,953–26,395 and 25,382–25,810, respectively. At a level of significance of p < 0.05, 1961 genes were significantly upregulated in 85As2 cells compared to in MKN45cl85 cells, while 2378 genes were downregulated. The resulting expression ratio (log2 ratio) data and Agilent Probe ID were imported into Ingenuity Pathways Analysis (IPA) ® software. A total of 1614 probes showed increased expression, whereas 1864 probes showed reduced expression of genetic information present in the IPA database. There were 1324 and 1607 unique genes displaying increased and decreased expression, respectively. The top five functions of genes with increased or decreased expression in different categories are shown in Tables 1 and 2. The top three categories showing increased expression starting from the highest degree of contribution were Disease and Disorders; Hereditary Disorders (135 molecules), Organismal Injury and Abnormalities (652 molecules), Dermatological Diseases and Conditions (177 molecules), Molecular and Cellular Functions; Cellular Movement (336 molecules), Cell-To-Cell Signaling and Interaction (266 molecules), and Cellular Growth and Proliferation (490 molecules). Categories showing decreased expression included Disease and Disorders; Cancer (1,488 molecules), Gastrointestinal Disease (1,301 molecules), Organismal Injury and Abnormalities (1,506 molecules), Molecular and Cellular Functions; Cellular Development (203 molecules), Cellular Growth and Proliferation (165 molecules), and Lipid Metabolism (88 molecules).

**Table 1:** Functional classification of genes with increased expression (the top five functions are shown for each category)

Category	Name	p-value range	Molecules
**Diseases and Disorders**			
	Hereditary Disorder	1.03E-02 - 2.67-E07	135
	Organismal Injury and Abnormalities	1.19E-02 - 2.67E-07	652
	Dermatological Diseases and Conditions	8.94E-03 - 4.66E-06	177
	Cancer	1.19E-02 - 8.85E-06	424
	Tumor Morphology	9.12E-03 - 8.85E-06	71
**Molecular and Cellular Functions**			
	Cellular Movement	1.12E-02 - 3.87E-07	336
	Cell-To-Cell Signaling and Interaction	1.18E-02 - 1.91E-06	266
	Cellular Growth and Proliferation	1.19E-02 - 1.91E-06	490
	Cell Signaling	6.73E-03 - 5.62E-05	110
	Cellular Assembly and Organization	9.90E-03 - 9.55E-05	239
**Physiological System Development and Function**			
	Hematological System Development and Function	1.06E-02 - 1.91E-06	200
	Immune Cell Trafficking	1.04E-02 - 1.05E-05	185
	Cardiovascular System Development and Function	1.19E-02 - 5.66E-05	145
	Tissue Morphology	1.17E-02 - 5.66E-05	116
	Organismal Functions	6.73E-03 - 2.05E-04	39

The resulting expression ratio (log2 ratio) data and Agilent Probe ID were imported into IPA® software. The number of unique genes with increased expression was 1324. The top five functions in different categories with functional analysis of genes with increased expression are shown.

**Table 2:** Functional classification of genes with decreased expression, with the top five functions shown for each category

Category	Name	p-value range	Molecules
**Diseases and Disorders**			
	Cancer	4.06E-02 - 4.34-E06	1488
	Gastrointestinal Disease	4.06E-02 - 4.34-E06	1301
	Organismal Injury and Abnormalities	4.06E-02 - 4.34-E06	1506
	Neurological Disease	4.06E-02 - 2.00E-04	328
	Ophthalamic Disease	3.46E-03 - 2.00E-06	10
**Molecular and Cellular Functions**			
	Cellular Movement	4.06E-02 - 4.94-E05	203
	Cellular Growth and Proliferation	4.06E-02 - 4.94-E05	165
	Lipid Metabolism	4.06E-02 - 2.98-E04	88
	Molecular Transport	4.06E-02 - 2.98-E04	128
	Nucleic Acid Metabolism	2.90E-02 - 2.98-E04	21
**Physiological System Development and Function**			
	Embryonic Development	4.06E-02 - 4.94-E05	185
	Hematological System Development and Function	4.06E-02 - 4.94-E05	42
	Hematopoiesis	4.06E-02 - 4.94-E05	33
	Lymphoid Tissue Structure and Development	4.06E-02 - 4.94-E05	26
	Organ Development	4.06E-02 - 4.94-E05	66

The resulting expression ratio (log2 ratio) data and Agilent Probe ID were imported into IPA® software. The number of unique genes with decreased expression was 1607. The top five functions in different categories with functional analysis of genes with decreased expression are shown.

**Figure 4:**
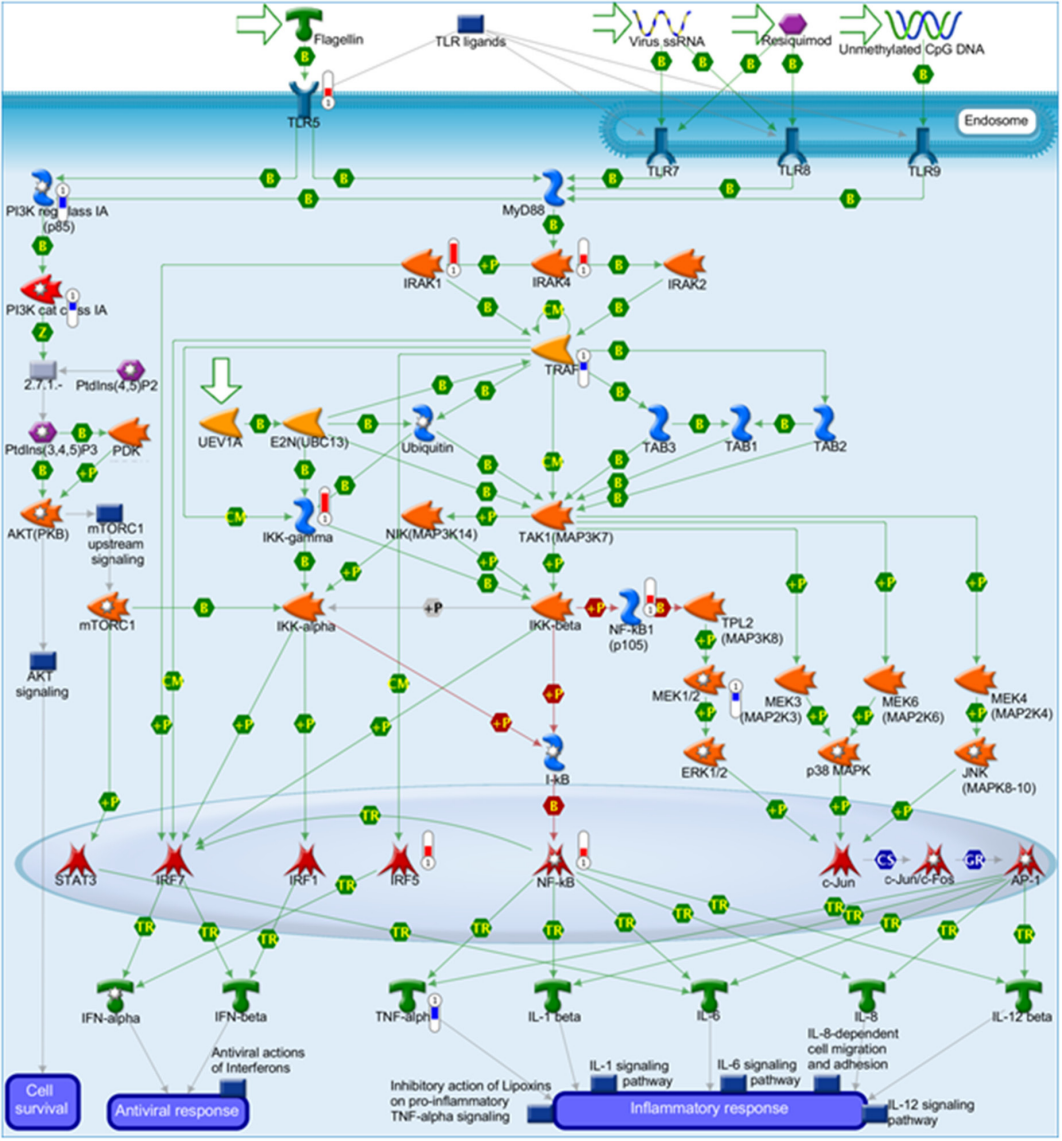
Increased gene expression of Toll-like receptor (TLR) 5 signaling in 85As2 cells compared to in MKN45cl85 cells The increased gene expression of the TLR signaling pathway in 85As2 cells compared to MKN45cl85 cells was demonstrated by MetaCore analysis of the DNA microarray. Red represents increased gene expression and blue represents decreased gene expression in 85As2 cells compared to MKN45cl85 cells.

The top five canonical pathways affected by the increased or decreased expression are listed in Table 3. The pathway most affected by increased expression was LPS/IL-1-mediated inhibition of retinoid X receptor (RXR) function (33 molecules) (Supplementary Figure 2). The pathway most affected by decreased expression was cholesterol biosynthesis I-III (seven molecules). Furthermore, we confirmed through IPA or MetaCore enrichment or pathway analysis that cytoskeleton remodeling-related pathways, TLR3/4 signaling, TLR 5/7/8/9 signaling, and nuclear factor-kappa B (NF-κB) activation were enriched. Additionally, Pathway Studio analysis also showed that the TLR4/5/7/9-NF-κB signaling was activated. Of these pathways, the TLR 5/7/8/9 signaling image map with MetaCore analysis is shown in Figure 4. In the TLR5 signaling pathway, increased expression of TLR5, interleukin-1 receptor-associated kinase (IRAK)-1, IRAK-4, and NF-κB were evident. Based on the increased expression of TLR5 (Figure 4) and CD 14 (Supplementary Figure 2), subsequent experiments focused on TLR4 or TLR5 signaling.

**Table 3:** Top five canonical pathway ranking

Gene expression	Name	p-value	Overlap
**Increased expression**			
	LPS/IL-1 Mediated Inhibition of RXR Function	9.38E-05	16.4% (33/200)
	Atherosclerosis Signaling	1.49E-04	18.7% (23/123)
	Eicosanoid Signaling	1.81E-03	20.3% (13/64)
	N-acetylglucosamine Degradation II	2.07E-03	75.0% (3/4)
	Fatty Acid α-oxidation	2.36E-03	33.3% (6/18)
**Decreased expression**			
	Cholesterol Biosynthesis I	5.02E-05	53.8% (7/13)
	Cholesterol Biosynthesis II (via 24,25-dihydrolanosterol)	5.02E-05	53.8% (7/13)
	Cholesterol Biosynthesis III (via Desmosterol)	5.02E-05	53.8% (7/13)
	Zymosterol Biosynthesis	8.50E-04	66.7% (4/6)
	Superpathway of Cholesterol Biosynthesis	1.52E-03	30.8% (8/26)

The resulting expression ratio (log2 ratio) data and Agilent Probe ID were imported into IPA® software. The number of unique genes with increased expression was 1324 and the number of unique genes with decreased expression was 1607. The top five canonical pathways affected by increased or decreased expression are listed. “Overlap” shows the genes (total) known to be involved in each pathway. The data from this analysis shows the proportion (%) of genes that exhibited increased or decreased expression in each pathway.

### Effects of TLR4 and of TLR5 stimulation on LIF production

**Figure 5:**
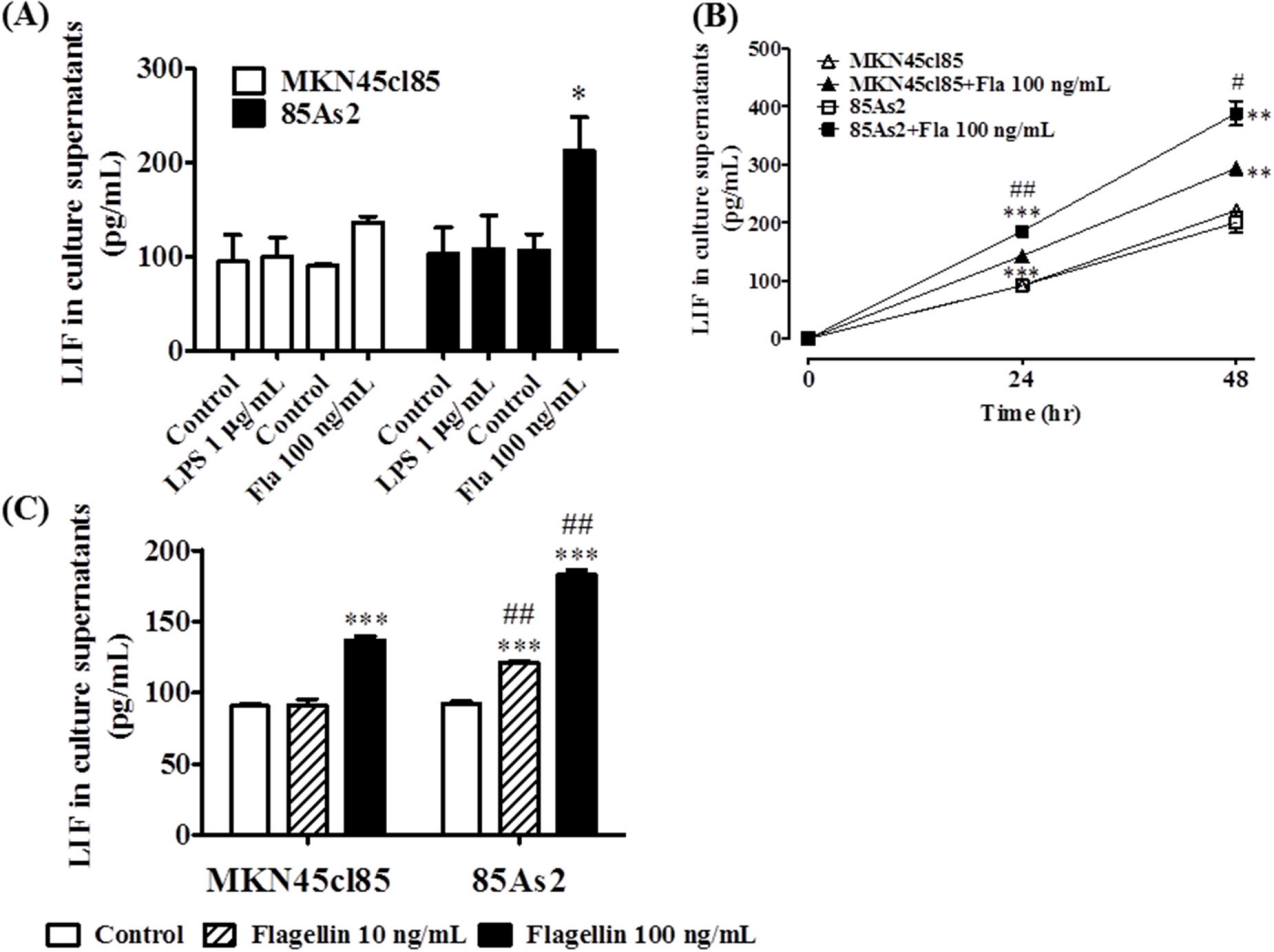
**(A)** Production of LIF by TLR4 or TLR5 ligand stimulation in MKN45cl85 or 85As2 cells. Both cell lines were treated with LPS (a TLR4 agonist) or flagellin (a TLR5 agonist) for 24 h. **(B)** Time course of LIF production by TLR5 ligand stimulation in MKN45cl85 or 85As2 cells. Both cell lines were treated for up to 48 h with flagellin (100 ng/mL). **(C)** Comparison between MKN45cl85 and 85As2 based on enhanced LIF production by flagellin. Both cells were seeded at 2.5 × 10^5^ cells/well in a 12-well plate and treated with flagellin at 10 or 100 ng/mL for 24 h. The levels of human LIF were measured in cell culture supernatants from the MKN45cl85 or 85As2 cell line. LIF levels are expressed as the mean ± SEM (pg/mL) values for three experiments (A) or three-six wells (B, C). Differences between the groups were evaluated using the Aspin–Welch's *t*-test. ^*^p < 0.05, ^**^p < 0.01, ^***^p < 0.001 vs. the corresponding control group (no TLR ligand); ^#^p < 0.05, ^##^p < 0.01 vs. the corresponding MKN45cl85 group (B, C); Fla: flagellin; LIF: leukemia inhibitory factor; LPS: lipopolysaccharide; TLR: Toll-like receptor.

To investigate whether TLR4 and TLR5 ligands promote LIF production in MKN45cl85 and 85As2 cells, the cells were treated with LPS (a TLR4 agonist) or flagellin (a TLR5 agonist) for 24 h, and the LIF concentration in the cell culture supernatants was measured. These cells produced approximately the same amount of LIF when not stimulated. Interestingly, LIF production was increased following addition of flagellin (100 ng/mL). 85As2 cells contained a significantly higher LIF concentration than MKN45cl85 cells, and a significant increase in LIF production was observed (Figure 5A). In contrast, LPS (1 μg/mL) did not affect LIF production by either cell type, even at a high concentration of 10 μg/mL (data not shown).

Next, we compared LIF production in these cells for up to 48 h after adding flagellin (100 ng/mL). LIF production significantly increased in both the MKN45cl85 and 85As2 groups at 24 and 48 h after adding flagellin compared to the unstimulated control (Figure 5B). The 85As2 group showed a particularly large increase in LIF production after flagellin addition; the increase was significantly higher than that in the MKN45cl85 group.

The addition of a low concentration of flagellin (10 ng/mL) did not affect MKN45cl85 cells, while LIF production in 85As2 cells was significantly increased compared to the unstimulated control and MKN45cl85 cells (Figure 5C). At a high concentration of flagellin (100 ng/mL), LIF production increased in MKN45cl85 cells and more prominently and significantly in 85As2 cells.

### Effects of TLR4 and of TLR5 stimulation on cell proliferation

**Figure 6:**
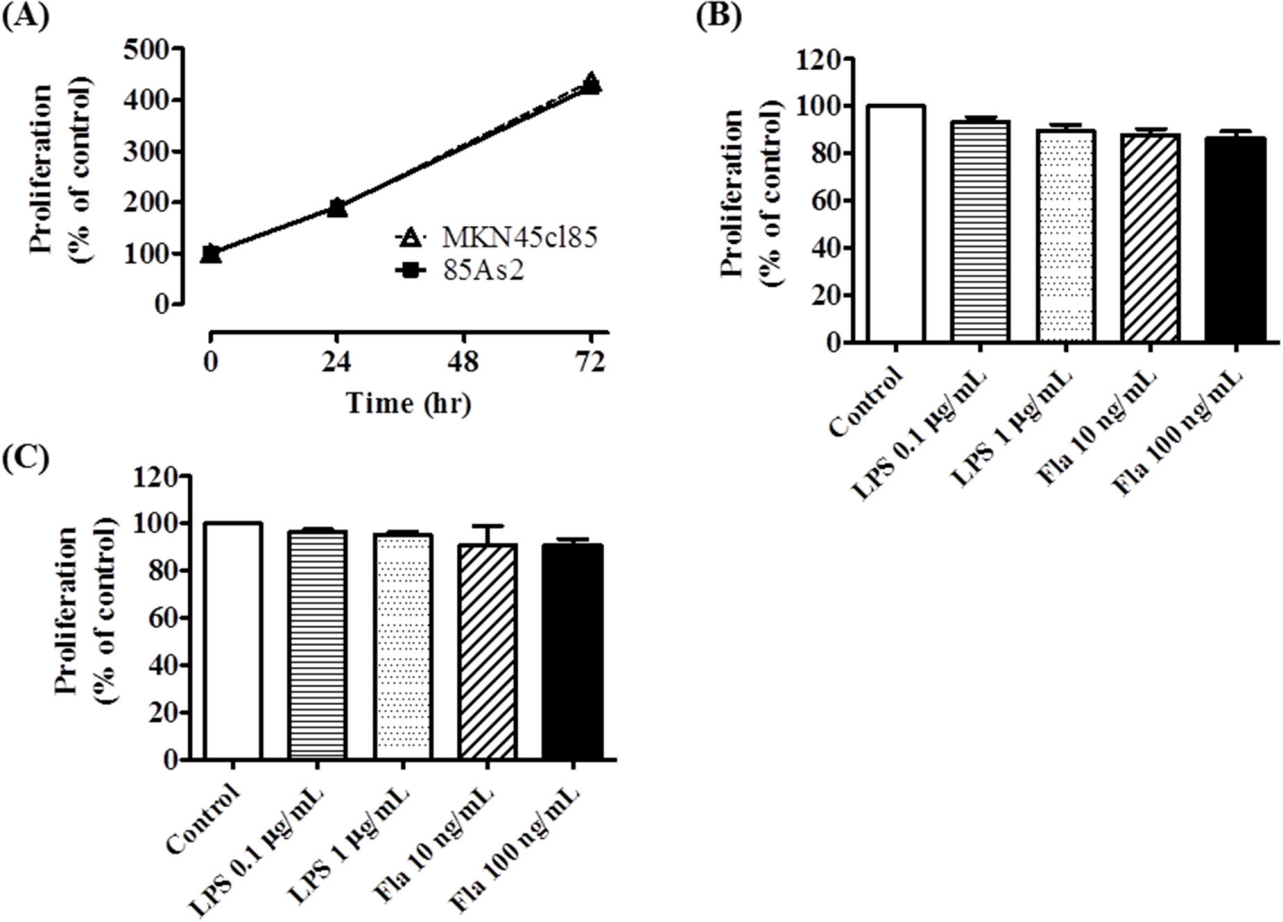
Effects of TLR4 or TLR5 ligand stimulation on cell proliferation in MKN45cl85 or 85As2 cells **(A)** Time course change in cell proliferation without TLR ligand stimulation. Each data point is expressed as a % of control at 0 h. The effect of LPS (a TLR4 ligand) or flagellin (a TLR5 ligand) on cell proliferation in **(B)** MKN45cl85 and **(C)** 85As2 cells. Both cells were seeded at 4 × 10^3^ cells/well in a 96-well plate and treated with LPS (0.1, 1 μg/mL) or flagellin (10, 100 ng/mL) for 72 h. The data are expressed as % of control, which is each value of the vehicle at 72 h. Each data point or column is expressed as the mean ± SEM for the six different values.MKN45cl85 or 85As2 cells were seeded at 4 × 10^3^ cells/well in a 96-well plate. Fla: flagellin; LPS: lipopolysaccharide; TLR: Toll-like receptor.

MKN45cl85 and 85As2 cell proliferation was observed for 72 h. The cells were seeded at 4 × 10^3^ cells/well at the start of culture and the cells proliferated at approximately the same rate over 72 h (Figure 6A). After 72 h, the proliferation of both cell types was more than 4-fold higher than the starting amount (% of control is the % of cells at time 0). There was no significant difference between the two cell types.

To determine whether TLR4 and TLR5 ligands promote MKN45cl85 and 85As2 cell proliferation, we added LPS or flagellin and evaluated cell proliferation after 72 h. No significant difference in cell proliferation was observed between either cell type in groups with added LPS (0.1, 1 μg/mL) and flagellin (10, 100 ng/mL) compared to the unstimulated control group (Figure 6B and 6C).

### Increased TLR5, IRAK-1/4 gene expression and inhibited TLR5 agonist-induced LIF production in 85As2 cells by IRAK-1/4 inhibitor

**Figure 7:**
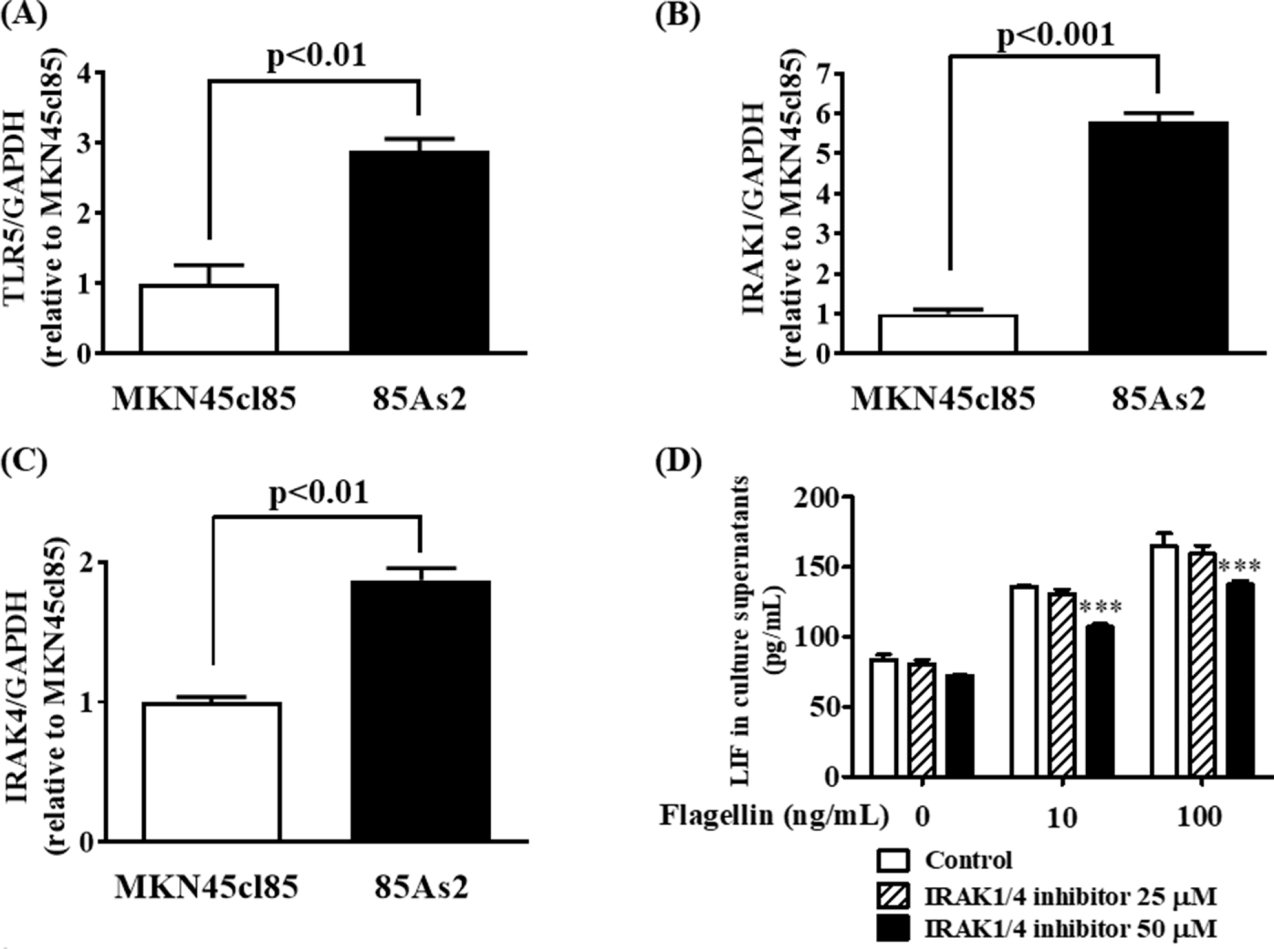
Enhancement of LIF secretion by stimulation of a TLR5 ligand via IRAK-1 and -4 in 85As2 cells Expression of **(A)** TLR5, **(B)** IRAK-1 and **(C)** IRAK-4 mRNA in MKN45cl85 and 85As2 cells. **(D)** Inhibitory effects of the IRAK-1/4 inhibitor on the enhancement of LIF secretion by a TLR5 ligand (flagellin) in 85As2 cells. 85As2 cells were stimulated with flagellin (10, 100 ng/mL) for 24 h in the presence or absence of an IRAK-1/4 inhibitor (25 or 50 μM). Each column represents the mean ± SEM of different values of three to six. Differences between the groups were evaluated using the Aspin–Welch's *t*-test (A, B, C) and using two-way repeated-measures analysis of variance followed by post-hoc Bonferroni tests (D). ^***^p < 0.001 vs. the vehicle (0.1% DMSO) group (D). DMSO: dimethyl sulfoxide; GAPDH: glyceraldehyde-3-phosphate dehydrogenase; IRAK: interleukin-1 receptor-associated kinase; LIF: leukemia inhibitory factor; TLR: Toll-like receptor.

DNA microarray analysis demonstrated that the expression of TLR5, IRAK-1, and IRAK-4, which are important in TLR5 signaling, was higher in 85As2 cells than in MKN45cl85 cells (Figure 4). To confirm this, we measured TLR5, IRAK-1, and IRAK-4 gene expression in MKN45cl85 and 85As2 cells. TLR5, IRAK-1, and IRAK-4 gene expression in 85As2 cells was increased compared to in MKN45cl85 cells; these differences between cell types were significant (Figure 7A, 7B and 7C). Next, we investigated the effect of an IRAK-1/4 inhibitor on LIF production in 85As2 cells. 85As2 cells produced LIF after culture for 24 h with no stimulation. Furthermore, LIF production of 85As2 cells increased in a concentration-dependent manner following flagellin addition (10, 100 ng/mL) (Figure 7D). In the presence of a higher concentration of an IRAK-1/4 inhibitor, LIF production was significantly suppressed for both 10 and 100 ng/mL flagellin, while this suppression was not observed in the unstimulated group.

### Increased TLR5, IRAK-1/4 gene expression in 85As2 cells-xenograft

**Figure 8:**
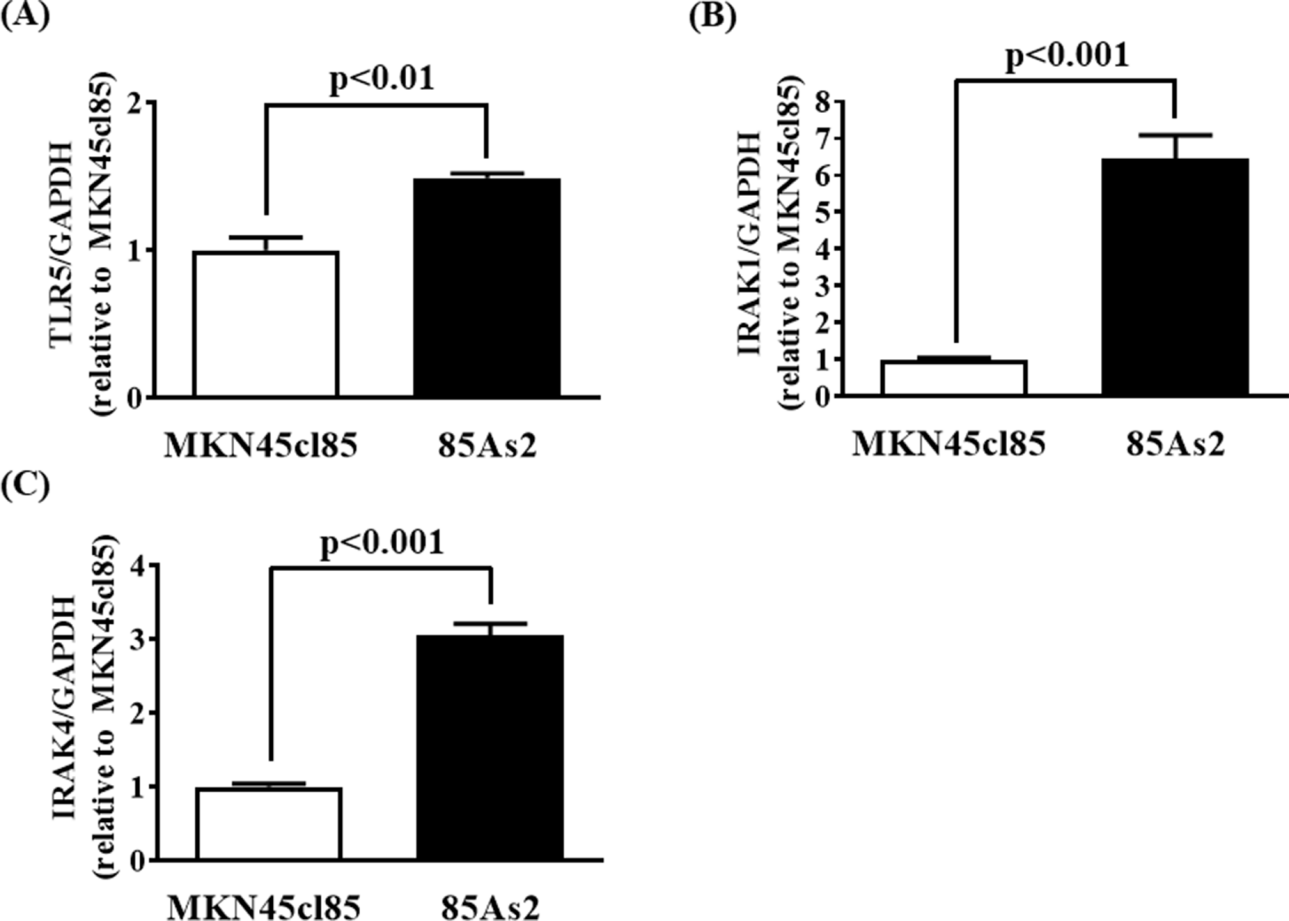
Enhanced expression of **(A)** TLR5, **(B)** IRAK-1, and **(C)** IRAK-4 mRNA in 85As2 cells-induced xenograft compared to that of MKN45cl85. Rats anesthetized by inhalation of 1–2.5% isoflurane were subcutaneously inoculated with 1 × 10^7^ cells at each site in the left and right flanks. After 2 weeks, the xenografts were carefully dissected. Each column represents the mean ± SEM of different values of five rats. Differences between the groups were evaluated using the Aspin–Welch's *t*-test. GAPDH: glyceraldehyde-3-phosphate dehydrogenase; IRAK: interleukin-1 receptor-associated kinase; LIF: leukemia inhibitory factor; TLR: Toll-like receptor.

To confirm whether the results of cell culture reflected *in vivo* conditions, we measured TLR5, IRAK-1, and IRAK-4 gene expression in tumor tissue induced by MKN45cl85 and 85As2 cells. TLR5, IRAK-1, and IRAK-4 gene expression in 85As2 cells was increased compared to that in MKN45cl85 cells; the differences between cell types were significant (Figure 8A–8C).

## DISCUSSION

**Figure 9:**
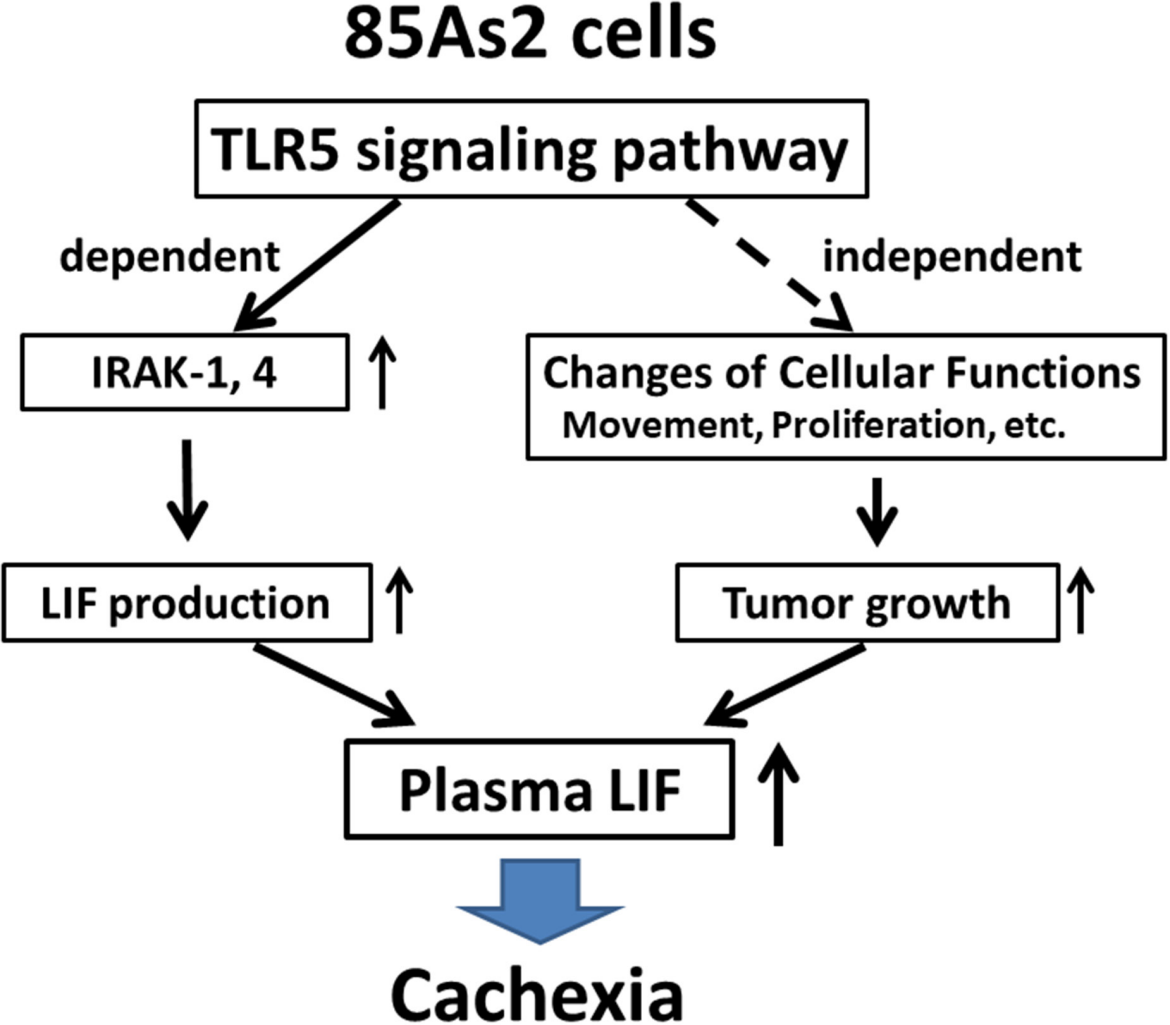
Schematic representation of the possible mechanism underlying the cachexia-inducing ability of 85As2 cells In 85As2 cells, the TLR5 signaling pathway is activated through increased IRAK-1/4 expression relative to that in the parent cell line MKN45cl85. Increased plasma LIF levels caused by increased LIF production through activation of the TLR5 signaling pathway may contribute to the onset or aggravation of 85As2 cell-induced cancer cachexia in rats. IRAK; interleukin-1 receptor-associated kinase; LIF: leukemia inhibitory factor; TLR: Toll-like receptor.

In this study, we found that in 85As2 cells, which were established from MKN45cl85 human gastric cancer cells, the TLR5 signaling pathway was activated with increased IRAK-1/4 expression compared to in the parent cell line. TLR5 stimulation promoted LIF production, suggesting that this mechanism contributes to activation of the cachexia-inducing ability of 85As2 cells (Figure 9). Additionally, the rapid development and enlargement of tumors also contributed to the onset or aggravation of cachexia in 85As2 cells. The promotion of tumor formation in 85As2 cells may be independent of TLR5 signaling. As separate mechanisms, it may be partly involved in altering cellular functions such as movement, growth, and proliferation *in vivo*.

Xenografts induced by 1 × 10^7^ MKN45cl85 cells induced symptoms of cachexia such as decreases in body weight and food consumption in a nude rat xenograft model. However, although tumors formed in rats receiving xenografts of 1 × 10^6^ MKN45cl85 cells, they did not exhibit symptoms of cachexia. However, 1 × 10^6^ 85As2 cells induced cachexia symptoms. Unexpectedly, the symptom intensity was similar to that caused by 1 × 10^7^ MKN45cl85 cells. Furthermore, 1 × 10^7^ 85As2 cell xenografts were more potent and induced symptoms of cachexia at earlier times. These results demonstrate that 85As2 cells have a greater ability to induce cachexia with greater severity than MKN45cl85 cells in a nude rat xenograft model. Our previous study suggested that LIF contributes to inducing cachexia in 85As2-induced cachexia model rats [8]. Therefore, we investigated plasma LIF concentrations to clarify the underlying mechanism of the difference in the cachexia-inducing ability of these cells. Plasma LIF levels were significantly higher in 85As2 cell groups than in MKN45cl85 cell groups after 4 weeks using xenografts containing 1 × 10^6^ and 1 × 10^7^ cells. Plasma LIF concentration after 4 weeks was inversely correlated with both muscle mass (R^2^ = 0.7311) and food intake (R^2^ = 0.7557), suggesting the higher LIF levels correlated with the severity of cachexia. On the other hand, the plasma LIF concentration in the MKN45cl85 1 × 10^7^ cell xenograft group was quite low, regardless of whether the cachexia symptoms were induced. Therefore, cachexia-inducing factors other than LIF may contribute to the disease induced by MKN45cl85 cells. Previous research showed that MKN45cl85 cells do not produce the cachexia-inducing factors IL-1β, IL-6, and TNF-α. LIF belongs to the IL-6 family. IL-6 family cytokines other than LIF, which include IL-6, IL-11, and CNTF, were also reported as cachexia-inducing factors [27–31]. Thus, we investigated whether MKN45cl85 cells produce these cytokines. MKN45cl85 and 85As2 cells only produced LIF, but did not produce IL-6, IL-11, or CNTF. Unexpectedly, unlike plasma LIF levels, there was no significant difference in LIF production by cultured cells between the two cell lines. The mechanism of the onset of cachexia due to MKN45cl85 cells is currently unclear. Our data suggest that only LIF contributes to cachexia onset caused by 85As2 cells, with no contribution by other IL-6 family cytokines.

DNA microarray analysis revealed differences in the gene expression levels of the two cell types. Changes were observed in the expression of genes related to cellular functions such as cellular movement, cell-to-cell signaling and interactions, and cellular growth and proliferation, as well as those related to cancer and tumor morphology. IPA analysis revealed fluctuations in the expression of genes in the canonical pathway for LPS/IL-1-mediated inhibition of RXR function. MetaCore and Pathway Studio analyses indicated activation of the TLR4 and TLR5 signaling pathways in 85As2 cells. Therefore, we evaluated the effects of TLR4 and TLR5 stimuli on cell proliferation and LIF production. These treatment groups showed approximately the same LIF production under unstimulated conditions. TLR5 stimulation by flagellin promoted LIF production in both cell types, but LIF production was much higher in 85As2 cells. Furthermore, a low concentration of flagellin did not promote LIF production in MKN45cl85 cells promoted LIF production in 85As2 cells. These results demonstrated increased TLR5 ligand sensitivity of 85As2 cells and that a TLR5 stimulus promotes LIF production. In contrast, IPA analysis revealed changes in LPS-related canonical pathways, while stimulation of TLR4 by LPS did not affect LIF production in either MKN45cl85 or 85As2 cells. These observations suggest that TLR5 signaling, not TLR4 signaling, promotes LIF production in 85As2 cells. DNA microarray analysis revealed increased mRNA expression of IRAK-1 and IRAK-4 in 85As2 cells compared to MKN45cl85 cells. The increased LIF production by TLR5 stimulation was significantly suppressed by an IRAK-1/4 inhibitor in 85As2 cells. This also supports the promotion of LIF production via activation of TLR5 signaling in 85As2 cells. Furthermore, in 85As2-induced xenografts, TLR5, IRAK-1, and IRAK-4 gene expression was significantly increased compared to that in MKN45cl85 cells, reflecting the characteristics of cell culture. These results suggest that activation of the TLR5 signaling pathway through elevated IRAK-1/4 expression contributes to promoting LIF production and worsening of cancer cachexia in 85As2 cells. This is the first study to report that LIF production is promoted by TLR5 in cancer cells as a mechanism underlying the worsening of cancer cachexia. Further studies of whether TLR5 or IRAK-1/4 antagonists improve cancer cachexia symptoms are necessary. These studies may provide evidence for the increased malignancy of cancer cells from peritoneal metastases.

TLR4 and TLR5 stimulation reportedly activate cancer cell proliferation and promote tumor formation by various cancer cells [16, 18, 30–32]. However, in the present study, TLR4 and TLR5 stimulation did not influence cell proliferation in either MKN45cl85 or 85As2 cells. After cancer cells are subcutaneously xenotransplanted, the resulting tumor tissue includes various cells other than the cancer cells themselves such as fibroblasts, leucocytes, and bacteria, which may be TLR ligands. In a preliminary study using luciferase-tagged MKN45cl85 and 85As2 cells, the proliferation of 85As2 cells in the tumor tissue was higher than the proliferation of MKN45cl85 cells, in addition to the enlarged tumor volume. This is consistent with the elevated plasma LIF concentration in rats with xenografts of either cell type and is related to the cachexia-inducing ability of the cells. DNA microarray analysis revealed changes in the expression of genes related to cellular functions such as cellular movement, cell-to-cell signaling and interactions, and cellular growth and proliferation. Thus, a separate mechanism is thought to contribute to the promotion of tumor formation by 85As2 cells.

In conclusion, 85As2 cells showed multiple genetic changes compared to their parent strain MKN45cl85, and these changes activated the TLR5 signaling pathway. LIF concentration in the plasma was increased by activation of the TLR5 signaling pathway; this suggests that LIF production contributes at least partially to activating the cachexia-inducing ability of 85As2 cells. This clarifies the understanding of the mechanism of the onset or aggravation of cachexia, which may be useful for developing treatments targeting peritoneal metastatic cancer cells.

## MATERIALS AND METHODS

### Animal experiments

Six-week-old male F344/NJcl-rnu/rnu rats (Clea-Japan, Tokyo, Japan) were housed individually using a 12:12-h light-dark cycle (lights on at 08:00 AM) at a constant temperature and humidity with *ad libitum* access to food and water. Rats were acclimated to laboratory conditions for 2 weeks prior to experimentation. All studies were approved by the Committee for Ethics in Animal Experimentation of the National Cancer Center and performed according to the Guidelines for Animal Experiments drafted by the committee (Approval Nos. T09-050-M02 and T09-050-C04). The experiments met the ethical standards required by the law and the guidelines concerning experimental animals in Japan.

### Cell lines, culture conditions, and reagents

MKN45cl85 and 85As2 cell lines were established from the human gastric MKN-45 cancer cell line as described previously [7]. Cells were maintained in RPMI 1640 medium (Nacalai Tesque, Inc., Kyoto, Japan) supplemented with 10% fetal bovine serum (Invitrogen, Carlsbad, CA, USA), 100 IU/mL penicillin G sodium, and 100 μg/mL streptomycin sulfate (Nacalai Tesque, Inc.) in a 5% CO_2_ atmosphere at 37°C. 3-(4, 5-Dimethylthiazol-2-yl)-2, 5-diphenyltetrazolium bromide (MTT) was purchased from Nacalai Tesque, Inc. LPS was purchased from Sigma Chemical (St. Louis, MO, USA). Flagellin was purchased from Enzo Life Sciences, Inc. (Farmingdale, NY, USA). IRAK-1/4 inhibitor was purchased from Merck Millipore (Billerica, MA, USA).

### Cancer cachexia rat model induced by implantation of MKN45cl85 or 85As2 cells

MKN45cl85 and 85As2 cells were harvested from subconfluent cultures after brief exposure to 0.25% trypsin and 0.2% ethylenediaminetetraacetic acid (EDTA). Cells were washed once in serum-free medium and resuspended in phosphate-buffered saline (PBS). Rats anesthetized by inhalation of 1–2.5% isoflurane (Mylan, Osaka, Japan) were subcutaneously inoculated with either 1 × 10^6^ or 10^7^ cells at each site (tumor-bearing rats) or with saline alone (non-tumor-bearing control rats) in the left and right flanks, respectively, at week 0. The major and minor tumor axes were measured, and tumor volume was estimated using the following equation: tumor volume (cm^3^) = major axis (cm) × minor axis (cm) × minor axis (cm) × ½ [33]. Body weight and food consumption were measured weekly in each model and expressed as the daily average. Plasma collected from the abdominal aorta was centrifuged (1700 ×*g* for 10 min) and stored at −80°C until analysis. Muscle (greater pectoral, gastrocnemius, tibialis, and soleus) and fat (epididymis, perirenal, and mesentery) tissues were immediately dissected and weighed.

### Cytokine measurements in cell culture supernatants

Human cytokine levels were measured in MKN45cl85 and 85As2 cell culture supernatants (2.5 × 10^5^ cells/well) at 24 and 48 h in a 12-well plate (BD Biosciences, Franklin Lakes, NJ, USA). Human IL-6, IL-11, or CNTF levels were measured using commercial enzyme-linked immunosorbent assay kits (Quantikine, R&D Systems, Inc., Minneapolis, MN, USA) with a minimum detectable dose of 8.00 pg/mL. Human LIF levels in the cell culture supernatant were measured using the Procarta® Cytokine Assay Kit (Affymetrix, Santa Clara, CA, USA). The minimum detectable dose was 2.44 pg/mL.

### Measurements of LIF levels in plasma or cell culture supernatants after stimulation with TLR4 or TLR5 agonist

To evaluate the effects of either a TLR4 or TLR5 agonist on the production of human LIF, MKN45cl85 or 85As2 cells were seeded at 2.5 × 10^5^ cells/well in 12-well plates (BD Biosciences). Both cell lines were cultured with or without LPS (a TLR4 agonist) or flagellin (a TLR5 agonist) at different concentrations (LPS: 0.1, 1 μg/mL; flagellin: 10, 100 ng/mL) for 24 h. Human LIF levels in cell culture supernatants were measured using the Procarta® Cytokine Assay Kit (Affymetrix). The minimum detectable dose was 2.44 pg/mL. Human LIF levels in the plasma of non-tumor-bearing or tumor-bearing rats also were measured using the same kit.

### DNA microarray analysis

MKN45cl85 and 85As2 cells were cultured in a 10-cm dish until they were semi-confluent and then removed from the dish by exposure to 0.25% trypsin and 0.2% EDTA for a short time. After centrifugation, total RNA was extracted with an RNeasy Mini kit (Qiagen, Hilden, Germany) according to the manufacturer's extraction protocol. Three RNA samples from cells cultured on different days were prepared (n = 3). RNA quality tests, microarray analyses, and IPA analyses were performed at the Chemical Evaluation and Research Institute (Saitama, Japan, http://www.cerij.or.jp/ceri_en/). RNA concentration and quality were measured using an ND-1000 spectrophotometer (NanoDrop Technologies, Winooski, VT, USA) and a model 2100 Bioanalyzer with an RNA 6000 Pico kit (Agilent Technologies, Santa Clara, CA, USA) to confirm that the samples were suitable for microarray analysis. Microarray analysis was performed according to the recommended One-Color Microarray-Based Gene Expression Analysis protocol (ver 6.0, December 2009; Agilent) using the Whole Human Genome Array (4 × 44 K) ver 2.0 (product code: G4845A, Agilent). Microarray hybridization was conducted for 17 h at 65°C. After washing, microarray scanning was conducted using a DNA microarray scanner (Agilent). The acquired image data were digitized using the Feature Extraction ver 10.7.1.1 software (Agilent).

Array data were normalized and a significance test (level of significance: p < 0.05) was conducted using GeneSpring GX 11.5 (Agilent). Additionally, the expression ratio (log2 ratio) was calculated using data from MKN45cl85 cells as the denominator and data from 85As2 cells as the numerator. The data were analyzed using IPA® Version 31313283 software (Ingenuity Systems, https://www.qiagenbioinformatics.com/).

Array data have been submitted to the Gene Expression Omnibus accession number GSE113110.

Probes were categorized based on their expression increase (1,961 probes) and decrease (2,378 probes). A list of the Agilent Probe ID and expression ratios (log2 ratios) was imported into IPA® in an Excel format and analyzed using the Core/Single program. The results are presented as a genetic functional classification (functional analysis) and pathway ranking (canonical pathway analysis). Furthermore, enrichment and canonical pathway analysis were performed with MetaCore^TM^ (Clarivate Analytics (Japan) Co., Ltd., Tokyo, Japan) or Pathway Studio® 9.0 (Elsevier, Amsterdam, Netherlands).

### Quantitative reverse transcription polymerase chain reaction

To evaluate gene expression in cell culture, MKN45cl85 and 85As2 cells were harvested from subconfluent cultures after brief exposure to 0.25% trypsin and 0.2% EDTA. Cells were washed once in PBS and then total RNA was extracted. To evaluate gene expression in the tumor tissue, xenografts induced by MKN45cl85 or 85As2 cells were prepared. Rats anesthetized by inhalation of 1–2.5% isoflurane were subcutaneously inoculated with 1 × 10^7^ cells at each site in the left and right flanks. After 2 weeks, the xenografts were carefully dissected after removing the blood from the abdominal aorta of anesthetized rats. The xenograft was cut with scissors and a center piece was used for total RNA extraction. Total RNA was isolated using an RNAeasy Mini kit (Qiagen) according to the manufacturer's instructions. Real-time polymerase chain reaction (PCR) was performed as previously described [34]. Briefly, first-strand cDNA was reverse-transcribed from 2.5 μg of total RNA using the Capacity RNA-to-cDNA kit (Applied Biosystems, Foster City, CA, USA) in a final volume of 50 μL. Diluted cDNA (2 μL) was amplified in a rapid thermal cycler (LightCycler; Roche Diagnostics, Basel, Switzerland) using LightCycler 480 SYBR Green I Mastermix (Roche Diagnostics) and the following primers: TLR5, (forward) 5′-GGAAGATGTCGGAGCCTCAG-3′ and (reverse) 5′-TGGTCTCCCATGATCCTCGT-3′; IRAK-1, (forward) 5′-TGC CTG GTG TAC GGC TTC-3′ and (reverse) 5′-CTG AGG CCA GGA GAG AGG T-3′); IRAK-4, (forward) 5′-GTT CGG CTG GTT CTT CTG TC-3′ and (reverse) 5′-GGG TTT GTT CAT CTT CTA TTC CTG-3′; and glyceraldehyde-3-phosphate dehydrogenase (forward) 5′-GCT CTC TGC TCC TCC TGT TC-3′ and (reverse) 5′-ACG ACC AAA TCC GTT GAC TC-3′. PCR products were quantified using LightCycler 480 software to analyze the exponential phase of amplification and melting curve as recommended by the manufacturer. The amount of target mRNA in the experimental group relative to that in the control group was determined from the resulting fluorescence and threshold values (C_T_) using the 2^−ΔΔCT^ method [35].

### Cell proliferation assay

MKN45cl85 or 85As2 cells were seeded at 4 × 10^3^ cells/well in a 96-well plate (BD Biosciences). Both cell lines were cultured with or without LPS (a TLR4 agonist) or flagellin (a TLR5 agonist) at different concentrations (LPS: 0.1, 1 μg/mL, flagellin: 10, 100 ng/mL) for 72 h. To assess cell proliferation, MTT was dissolved in PBS at a final concentration of 5 mg/mL and then sterilized with a 0.2-μm filter. After culture, 10 μL of MTT solution was added to each well and incubated at 37°C for 4 h. The supernatant was removed, MTT formazan was dissolved in 100 μL dimethyl sulfoxide, and the samples were shaken for 15 min. Absorbance at 563 nm was measured with a microplate reader (Tecan, Männedorf, Switzerland) using wells without cells as blanks.

### Statistical analyses

All data are expressed as the mean ± standard error of the mean (SEM). Differences between groups were evaluated using the Aspin–Welch's *t*-test, Mann-Whitney U-test, or Kruskal-Wallis test followed by a post-hoc Dunn's multiple comparison test or two-way repeated-measures analysis of variance followed by post-hoc Bonferroni tests. The significance level α was set at 0.05, and differences with a two-sided *p*-value < 0.05 were considered significant. All statistical analyses were performed using GraphPad Prism version 5 (GraphPad Software, Inc., La Jolla, CA, USA).

## SUPPLEMENTARY MATERIALS FIGURES



## Abbreviations

CNTF: Ciliary neurotrophic factor
EDTA: ethylenediaminetetraacetic acid
GAPDH: glyceraldehyde-3-phosphate dehydrogenase
IL: interleukin
IPA: Ingenuity Pathways Analysis
IRAK: interleukin-1 receptor-associated kinase
LIF: leukemia inhibitory factor
LPS: lipopolysaccharide
PBS: phosphate-buffered saline
RXR: retinoid X receptor
TLR: toll-like receptor
TNF-α: tumor necrosis factor-alpha
