# Supplementary material for: Leukemia inhibitory factor via the Toll-like receptor 5 signaling pathway involves aggravation of cachexia induced by human gastric cancer-derived 85As2 cells in rats
Source: Oncotarget. 2018 Oct 5;9(78):34748–64. doi: 10.18632/oncotarget.26190 (PMC6205166; doi:10.18632/oncotarget.26190)
Supplement: Supplementary file 1 [file oncotarget-09-34748-s001.pdf]

Leukemia inhibitory factor via the Toll-like receptor 5 signaling pathway involves aggravation of cachexia induced by human gastric cancer-derived 85As2 cells in rats

SUPPLEMENTARY MATERIALS

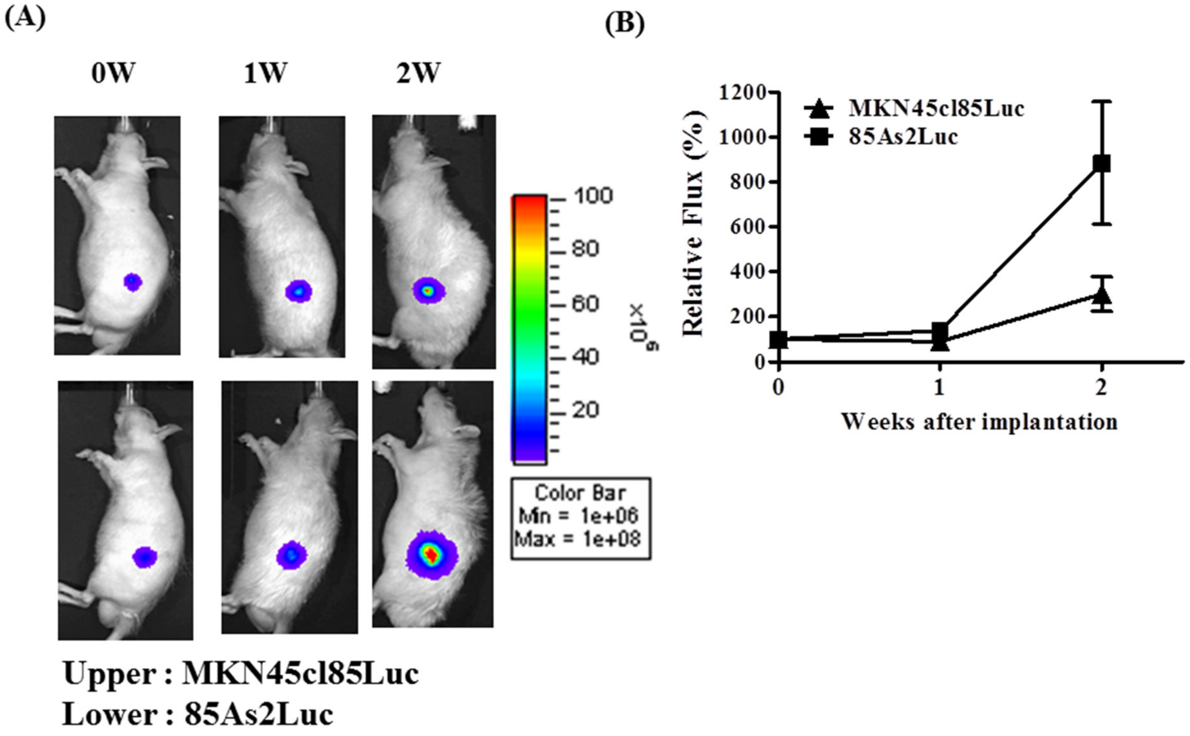

Supplementary Figure 1: Comparison of tumor growth between MKN45cl85- and 85As2-bearing nude rats. Both MKN45cl85 and 85As2 cells were transfected the luciferase gene with green fluorescence protein vectors; competent MKN45cl85 and 85As2 cells carrying the luciferase gene were designated as MKN45cl85Luc and 85As2Luc, respectively. Rats were inoculated subcutaneously with either MKN45cl85Luc or 85As2Luc cells in both flanks (1×10^7 cells at each site) at week 0. To assess the tumorigenicity of MKN45cl85Luc and 85As2Luc, bioluminescence signals from these cells implanted into rats were monitored after intraperitoneal injection of D-luciferin (150 mg/kg) using the *In Vivo* Imaging System Lumina series (Caliper Life Sciences, Waltham, MA, USA). During monitoring, the animals were kept under isoflurane anesthesia. The tumor volume was identified as the region of interest of bioluminescence and total photon counts were quantified using the Living Image acquisition and Analysis Software (Caliper Life Sciences). **(A)** Tumor sizes in rats is indicated by color (red is intense, while blue is the opposite) as a representative example. **(B)** Data of total photon counts were expressed as the % of week 0. Each data point represents the mean ± SEM of both sites of three rats (n = 6).

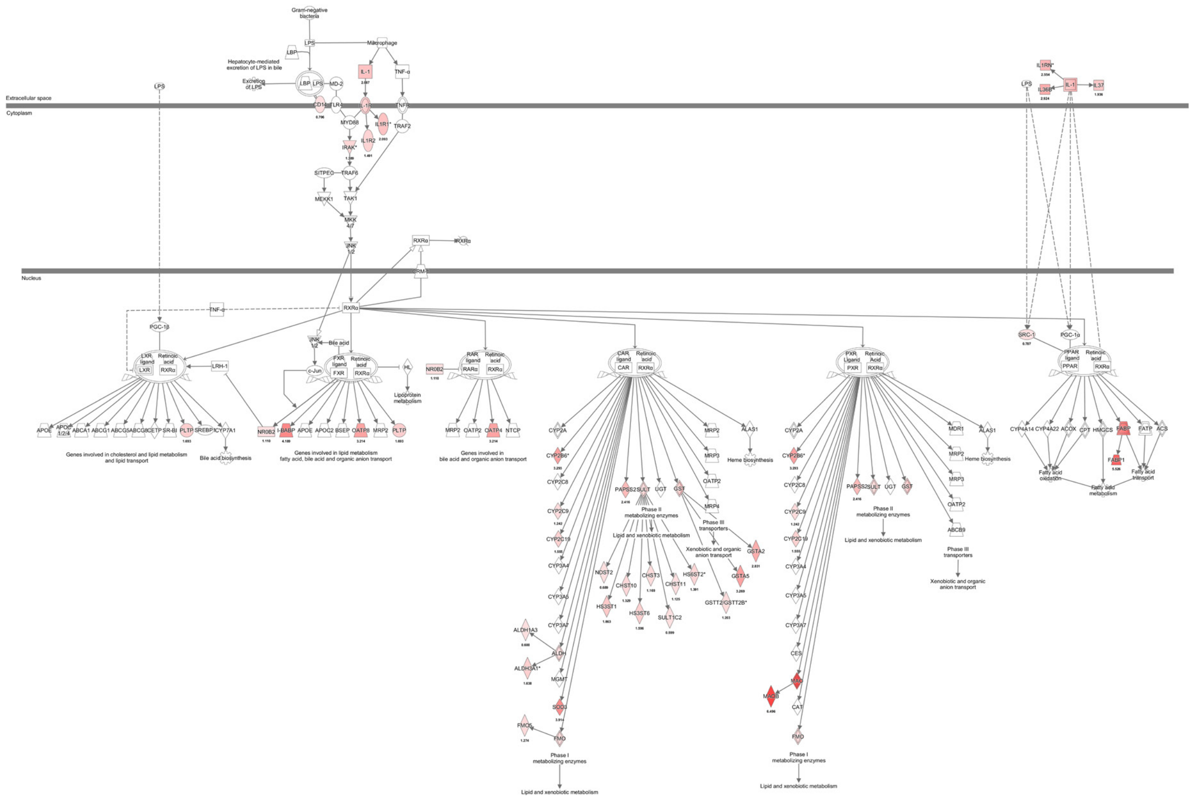

Supplementary Figure 2: LPS/IL-1 mediated inhibition of RXR function, which is the top canonical pathway affected by the increased gene expression when 85As2 cells were compared to MKN45cl85 cells. RXR: retinoid X receptor.
